# A healthy cities agenda for extreme heat adaptation in urban settings

**DOI:** 10.2471/BLT.25.294493

**Published:** 2026-01-14

**Authors:** Alaha Nasari, Sammer Marzouk

**Affiliations:** a FXB Center for Health and Human Rights, Harvard University, 651 Huntington Ave., Boston, MA 02115, United States of America (USA).; b Feinberg School of Medicine, Northwestern University, Chicago, USA

## Abstract

Extreme heat has become a predictable urban health emergency. Here, we synthesize evidence from 2024 and 2025 from peer-reviewed studies, multilateral agency reports and city heat-action plans on urban heat adaptation and health. Based on this evidence, we propose a city-level agenda grounded in the World Health Organization Healthy Cities framework. We outline interventions with demonstrated benefits, including urban greening and blue infrastructure (such as rivers, canals, wetlands and other urban water features that reduce ambient temperatures), cool roofs and reflective pavements, shaded pedestrian and transit corridors, as well as housing retrofits that prioritize passive cooling and equitable access to efficient cooling. We place equal emphasis on health-system readiness: impact-based early warning, surge protocols, clinician training, cooling centres and strengthened surveillance. We examine implementation challenges in financing, governance, equity, technical capacity and regulation, and propose practical city governance responses. We emphasize two contributions to urban heat adaptation policy and practice: hyperlocal targeting tools to maximize equity and effectiveness, and positioning health systems alongside infrastructure, rather than treating adaptation as a purely built-environment intervention.

## Résumé

Les vagues de chaleur sont devenues une urgence sanitaire prévisible en milieu urbain. Le présent article synthétise les données issues d’études évaluées par des pairs, de rapports d’agences multilatérales et de plans d’action contre la chaleur urbaine pour les années 2024 et 2025, qui portent sur l’adaptation à la chaleur et sur la santé. À partir de ces données, nous y formulons un programme à l’échelle des villes fondé sur le cadre politique des Villes-Santé de l’Organisation mondiale de la Santé. Nous décrivons également des interventions dont les avantages ont été démontrés, notamment la végétalisation urbaine et la trame bleue (constituée par les rivières, canaux, zones humides et autres éléments aquatiques urbains qui réduisent la température ambiante), les toits frais et les chaussées réfléchissantes, les couloirs ombragés pour piétons et transports en commun, ainsi que la rénovation de logements qui privilégie le refroidissement passif et l’accès équitable à un refroidissement efficace. À cet effet, nous accordons une importance égale à la préparation des systèmes de santé: alertes précoces basées sur l’impact, protocoles de masse, formation des cliniciens, centres de rafraîchissement et renforcement de la surveillance. Nous examinons par ailleurs les défis liés à la mise en œuvre en matière de financement, de gouvernance, d’équité, de capacités techniques et de réglementation et proposons des solutions pratiques en matière de gouvernance urbaine. Enfin, nous mettons l’accent sur deux contributions aux politiques et aux pratiques d’adaptation à la chaleur urbaine : des outils de ciblage hyperlocaux destinés à maximiser l’équité et l’efficacité, ainsi que le positionnement des systèmes de santé aux côtés des infrastructures, plutôt que de traiter l’adaptation comme une intervention purement liée à l’environnement bâti.

## Resumen

El calor extremo se ha convertido en una emergencia sanitaria urbana predecible. En este artículo, se sintetiza la evidencia de 2024 y 2025 procedente de estudios revisados por pares, informes de organismos multilaterales y planes de acción ante el calor de ciudades, sobre la adaptación al calor urbano y la salud. A partir de esta evidencia, se propone una agenda a nivel municipal basada en el marco de Ciudades Saludables de la Organización Mundial de la Salud. Se describen intervenciones con beneficios demostrados, como el reverdecimiento urbano y la infraestructura azul (como ríos, canales, humedales y otros elementos acuáticos urbanos que reducen las temperaturas ambiente), los techos fríos y los pavimentos reflectantes, los corredores peatonales y de transporte en sombra, así como la rehabilitación de viviendas que priorice el enfriamiento pasivo y el acceso equitativo a soluciones de refrigeración eficientes. Se otorga la misma importancia a la preparación del sistema de salud: alerta temprana basada en impactos, protocolos de aumento repentino de la demanda, capacitación del personal clínico, centros de enfriamiento y fortalecimiento de la vigilancia. Se analizan los desafíos de implementación en financiación, gobernanza, equidad, capacidad técnica y regulación, y se proponen respuestas prácticas de gobernanza municipal. Se destacan dos aportaciones a la política y la práctica de adaptación al calor urbano: herramientas de focalización hiperlocal para maximizar la equidad y la eficacia, y la incorporación de los sistemas de salud junto con la infraestructura, en lugar de tratar la adaptación como una intervención exclusivamente del entorno construido.

## ملخص

أصبحت الحرارة الشديدة حالة طوارئ صحية متوقعة في المناطق الحضرية. في هذا البحث، نلخص الأدلة من عامي 2024 و2025 من دراسات محكمة، وتقارير وكالات متعددة الأطراف، وخطط عمل المدن لمواجهة الحرارة، وذلك فيما يتعلق بالتكيف مع الحرارة والصحة في الأماكن الحضرية. بناءً على هذه الأدلة، نحن نقترح جدول للأعمال على مستوى المدينة في إطار عمل منظمة الصحة العالمية للمدن الصحية. نحن نحدد التدخلات ذات الفوائد المشهودة، بما في ذلك المساحات الخضراء الحضرية والبنية التحتية المائية (مثل الأنهار والقنوات والأراضي الرطبة وغيرها من المسطحات المائية الحضرية التي تخفض درجات الحرارة المحيطة)، والأسقف الباردة، والأرصفة العاكسة، وممرات المشاة والنقل المظللة، بالإضافة إلى تحديثات المساكن التي تعطي الأولوية للتبريد السلبي، والوصول عادل إلى أنظمة التبريد الفعالة. نحن نوجه اهتمامًا متساويًا لجاهزية النظام الصحي: الإنذار المبكر القائم على التأثير، وبروتوكولات الاستجابة السريعة، وتدريب الأطباء، ومراكز التبريد، وتعزيز المراقبة. وندرس تحديات التنفيذ في مجالات التمويل والحوكمة والإنصاف والقدرات التقنية والتنظيم، ونقترح استجابات عملية لحوكمة المدن. نحن نؤكد على مساهمتين في سياسة وممارسة التكيف مع الحرارة في المناطق الحضرية: أدوات الاستهداف شديدة المحلية لزيادة الإنصاف والفعالية إلى أقصى حد، ووضع النظم الصحية جنبًا إلى جنب مع البنية التحتية، بدلاً من التعامل مع التكيف كمجرد تدخل قائم على البيئة.

## 摘要

极端高温已成为可预测的城市公共卫生突发事件。我们在本文中综合分析了 2024 年和 2025 年有关城市高温适应和公共健康的经同行评审的研究、多边机构报告和城市高温行动计划的相关证据。。根据此类证据，我们建议基于世卫组织健康城市框架制定市级议程。我们概述了经证明具有显著效益的干预措施，包括城市绿化和蓝色基础设施（例如河流、运河、湿地及其他有助于降低环境温度的城市水景）、凉爽屋顶和高反射路面、带遮阳设施的行人和公共交通廊道以及以被动式降温为核心、保障公平使用高效降温设施的住房改造工程。我们认为卫生系统做好以下准备工作同等重要：基于影响的预警、应急增援预案、临床医生培训、降温中心和加强监测。我们分析了在筹资、治理、公平性、技术能力和监管方面存在的实施挑战，并建议制定切实可行的城市治理措施。我们强调从两个方面着手推行城市高温适应政策和实践：采用能够最大程度地保证公平性和有效性的超本地化精准靶向工具，以及将卫生系统与基础设施置于同等重要的战略地位，而不是将高温适应政策单纯地视为建成环境干预措施。

## Резюме

Экстремальная жара стала предсказуемым чрезвычайным фактором в городах. Авторы объединили данные из рецензируемых исследований за 2024 и 2025 годы, отчетов многосторонних агентств и городских планов по борьбе с жарой (адаптации городов и системы здравоохранения к условиям жары). Опираясь на эти данные, авторы предложили повестку дня на уровне городов, опирающуюся на рамочную политику Всемирной организации здравоохранения «Здоровые города». Авторы описывают действия, которые показали реальную пользу, включая озеленение городов и водную инфраструктуру (такую как реки, каналы, обводненные участки и другие городские водные структуры, которые снижают температуру окружающей среды), холодные крыши и отражающее дорожное покрытие, затененные пешеходные зоны и транспортные коридоры, а также модернизацию жилья с первоочередным вниманием к пассивному охлаждению и равному доступу к эффективному охлаждению. Равное внимание уделяется также готовности систем здравоохранения: раннему предупреждению на основе оценки воздействия, протоколам реагирования на пиковые нагрузки, обучению клиницистов, центрам охлаждения и усиленному надзору. Авторы анализируют ключевые проблемы реализации, связанные с финансированием, системой управления, обеспечением справедливости, техническими возможностями и нормативным регулированием, и предлагают практические управленческие решения на уровне городов. Особо выделены два важных направления в политике и практике адаптации городов к жаре: применение гиперлокальных инструментов, повышающих справедливость и эффективность, и интеграция систем здравоохранения наравне с инфраструктурными решениями, а не сведение адаптации лишь к изменениям застроенной среды.

## Introduction

Extreme heat has become a predictable public health emergency rather than an occasional anomaly.^1^ Between May 2024 and May 2025, nearly half of the world’s population experienced at least 30 days of extreme heat.^2^ Mortality data from the past two decades suggest that almost 500 000 annual deaths are attributable to heat, with the World Health Organization (WHO) European and South-East Asia Regions experiencing the highest burdens.^1^ Furthermore, heat also increases risks of kidney injury,^3^ cardiovascular strain,^4^ occupational hazards and productivity losses.^5^ Cities, with dense populations and built environments that amplify heat, face the highest risks but also hold the greatest potential for solutions. The WHO Healthy Cities framework emphasizes that health outcomes depend on coordinated action across housing, transport, education, environment and health system sectors.^1^ Applying this framework provides a structured way to align interventions across sectors, ensure equity and build resilience in the face of accelerating climate change.

## Evidence-based interventions

Cities worldwide are beginning to demonstrate how local policies can mitigate the health effects of extreme heat. In Paris, France, the Oasis Project transformed schoolyards into shaded, permeable and cooler spaces.^6^ Microclimatic analysis showed that these redesigned schoolyards reduced heat within school grounds and lowered temperatures in surrounding neighbourhoods, proving that relatively small-scale interventions can produce wider urban benefits.^6^^,^^7^ Similarly, Medellín’s green corridors in Colombia, which connect roads and waterways with trees and plants, achieved average temperature reductions of 2 °C in just three years while also creating jobs for disadvantaged populations.^8^ These cases illustrate how cities can redesign public spaces to simultaneously provide cooling and promote social equity.

Recent advances in modelling of urban thermal stress have strengthened the capacity of cities to target heat adaptation interventions. The Global Scale Model-Universal Thermal Climate Index framework, tested in 2025 in Philadelphia, United States of America, simulated thermal stress in small spatial units and demonstrated that converting impermeable surfaces to vegetation reduced heat stress by more than 4 °C.^9^ This highly localized approach allows city governments to prioritize neighbourhoods where public health gains will be greatest. Equity-focused analyses reinforce the need for such precision, showing that cities in low- and middle-income countries often have fewer green spaces and less access to cooling benefits than their counterparts in high-income countries.^1^ Without deliberate targeting, new infrastructure may widen rather than close these gaps.

Housing policies are another area of innovation. A recent study in New York City, United States, modelled indoor cooling needs among vulnerable populations under multiple warming scenarios.^10^ The study recommended combining subsidized access to efficient air conditioning with building envelope improvements such as insulation and reflective roofing.^10^ This dual approach balances immediate relief with long-term sustainability, while avoiding overreliance on energy-intensive cooling. In Ahmedabad, India, cool roof programmes in informal settlements have already demonstrated the value of reflective surfaces, by reducing indoor temperatures and lowering heat stress among residents.^11^ These examples highlight the potential for housing policy to directly reduce health risks.

Transport and planning strategies also influence exposure. Cities in the United States, including Los Angeles and Phoenix, have adopted reflective pavements, shaded walkways and cooler transit stops to reduce risk for pedestrians and transit users.^12^ Such measures are especially critical in underserved neighbourhoods where tree canopy is sparse and residents rely heavily on walking and public transport. The C40 Cool Cities Network^8^ has documented similar equity-driven initiatives in cities across the Region of the Americas and the African Region, where community engagement and vulnerability mapping guide the placement of cooling infrastructure in high-risk areas.^8^ These cases show that local action, when coordinated and evidence-based, can meaningfully reduce exposure to dangerous heat.

## The Healthy Cities framework

The WHO Health Cities framework provides a unifying approach to adaptation^1^ by recognizing that health is shaped by decisions across sectors. The framework ensures that heat interventions are not isolated or piecemeal. For example, expanding green space must be aligned with zoning codes that preserve airflow, housing regulations that guarantee access to cooling for all residents, and education campaigns on protective behaviours. Similarly, early warning systems must connect meteorological forecasts with health system protocols and targeted community outreach.^13^^,^^14^ The framework also prioritizes equity by promoting health and equitable development across urban policies, ensuring that city actions address the needs of underserved and vulnerable populations.^1^ Embedding heat adaptation in this model transforms climate action into a coordinated health strategy that links environment, governance, infrastructure and social policy.

## Infrastructure and health systems

Within this framework, infrastructure and health systems play complementary roles. Although infrastructure investments are important, health systems must be equally prepared to respond to extreme heat. Early warning systems that link weather forecasts to targeted health advisories have already reduced heat-related mortality in several cities,^15^ with evidence showing that heat warning systems can affectively lower deaths during extreme heat events. Hospitals need surge protocols to handle spikes in heat-related admissions, designated cooling areas to treat vulnerable patients and staff trained to distinguish heat illness from infectious disease. Medical schools have begun incorporating climate health into their curricula,^16^ yet transformation requires extending this knowledge into licensing requirements, continuing professional education and routine hospital practice.

Housing remains a decisive determinant of health risk.^1^ Informal and poorly insulated homes amplify indoor heat, creating dangerous conditions during prolonged heatwaves. Passive measures such as reflective roofs, shading and insulation reduce exposure, while efficient cooling devices provide additional relief.^10^^,^^11^ Policies must address energy poverty by subsidizing cooling for low-income households and preventing landlords from neglecting minimum cooling standards.^10^ Without such protections, adaptation risks deepening existing inequalities.

Transport and urban design also shape vulnerability. Shaded sidewalks, cooler transit stops and orientation of new buildings to preserve airflow all reduce exposure for urban residents.^12^ Because these interventions often fall under the purview of different city departments, coordination is critical to ensure consistency and equity.

Finally, public education and governance are essential to strengthen technical measures.^1^ Awareness campaigns can change behaviours that reduce heat risk, while participatory planning builds public trust and ensures interventions reflect community priorities. Emerging governance mechanisms, such as the appointment of chief heat officers and the creation of cross-departmental task forces, demonstrate how cities can institutionalize responsibility for heat adaptation.^17^

## Implementation challenges

Despite promising progress, considerable obstacles remain. Financial barriers are among the most pressing, as green infrastructure, housing retrofits and cooling centres demand large upfront investments and ongoing maintenance. Many cities, particularly in low- and middle-income countries, lack the fiscal capacity to sustain such programmes.^18^ Governance fragmentation further complicates implementation. Planning, housing, health and transport departments often operate in silos, leading to gaps and inconsistencies in adaptation strategies.^1^

Equity is another persistent challenge. Vulnerable populations are frequently located in the hottest neighbourhoods but have the least political influence and the fewest resources to adapt.^19^ Without deliberate prioritization, adaptation may reinforce rather than reduce disparities. Technical barriers, including a lack of granular climate and health data, limit the ability of city governments and planning authorities to identify hotspots and measure intervention outcomes.^20^Even where advanced modelling tools exist, limited technical expertise, staffing capacity and financial resources can restrict their use.^9^

Finally, political inertia slows progress. Adaptation projects often compete with short-term development priorities and face resistance from property developers or infrastructure service providers, such as energy or water utilities, whose incentives may not align with long-term adaptation goals. Building codes and housing regulations are slow to change, and public awareness of heat risks remains limited until a crisis occurs.^18^ These factors combine to delay the implementation of policies that could save lives.

## Policy agenda and contributions

Here, we propose a healthy cities heat adaptation agenda that integrates multiple domains under the WHO Healthy Cities framework. This agenda is directed at city governments and municipal decision-makers and is intended to guide coordinated action across planning, housing, transport and health system authorities. Data collection and vulnerability mapping will be essential in this framework because they can specifically target high-risk areas and inform specialized interventions.^9^ Built environment strategies should expand urban greening and blue spaces (including rivers, canals, wetlands and other urban water features that provide evaporative cooling), integrate these features into public space design, and implement reflective pavements and roofs alongside passive cooling requirements in building codes.^11^^,^^12^ Housing policy must include retrofits for low-income communities and subsidies for efficient cooling, while preventing energy poverty.^10^ Health systems should establish early warning systems, cooling centres and clinical training programmes.^14^^,^^16^ Transport and planning authorities must create shaded pedestrian corridors and cooler public transit infrastructure.^12^ Governance frameworks should institutionalize responsibility through city-level heat officers and cross-sector task forces.^17^ Policies must embed community engagement throughout to ensure cultural relevance and public trust.^8^

This agenda advances three novel contributions to existing calls for adaptation. First, it highlights the potential of locally focused predictive models to guide interventions with precision, maximizing health benefits and equity.^9^ Second, it emphasizes that adaptation must reduce average temperatures and guarantee access to cooling for vulnerable populations.^10^^,^^11^ Third, it places health systems at the centre of adaptation, expanding beyond education to include early warning, surveillance and emergency protocols.^14^^,^^21^ Together, these elements strengthen the case for treating extreme heat as an urgent and systemic health threat.^1^

## Conclusion

Extreme heat is a challenge for urban health in the twenty-first century, driving increased mortality and disease burden, and widening inequalities.^22^ Nevertheless, cities have the tools to act. By applying the WHO Healthy Cities framework, the healthy cities heat adaptation agenda proposed here translates this framework into coordinated action across infrastructure, housing, transport, health systems and governance.^1^ This agenda provides a practical pathway to ensure that adaptation is systematic, equitable and sustainable. While global mitigation of greenhouse gas emissions remains essential, city-level action guided by this agenda offers the most immediate protection for vulnerable populations.^18^
